# Involvement of adiponectin in early stage of colorectal carcinogenesis

**DOI:** 10.1186/1471-2407-14-811

**Published:** 2014-11-05

**Authors:** Chung Hyun Tae, Seong-Eun Kim, Sung-Ae Jung, Yang-Hee Joo, Ki-Nam Shim, Hye-Kyung Jung, Tae Hun Kim, Min-Sun Cho, Kwang Ho Kim, Joung Sook Kim

**Affiliations:** Department of Internal Medicine, Ewha Medical Research Institute, Ewha Womans University School of Medicine, Seoul, Korea; Department of Pathology, Ewha Medical Research Institue, Ewha Womans University School of Medicine, Seoul, Korea; Department of Surgery, Ewha Medical Research Institute, Ewha Womans University School of Medicine, Seoul, Korea; Department of Health Promotion Medicine, Ewha Medical Research Institute, Ewha Womans University School of Medicine, Seoul, Korea

**Keywords:** Adiponectin, Adiponectin receptors, Colorectal cancer, Carcinogenesis

## Abstract

**Background:**

Although altered levels of adiponectin have been reported as a potential risk factor in colorectal cancer (CRC), the importance of the role played by adiponectin in colorectal carcinogenesis has not been established. We sought to examine the expression pattern of adiponectin and adiponectin receptors (AdipoRs) in the normal-adenoma-carcinoma sequence and to assess the implications of adiponectin in colorectal carcinogenesis.

**Methods:**

Serum adiponectin concentrations, and the mRNA and protein expression of adiponectin and AdipoRs were examined using serum and tissues from patients with CRC, advanced adenoma, and a normal colon. mRNA expression of AdipoRs and epithelial-mesenchymal transition regulators including E-cadherin, cyclooxygenase-2 (COX-2) and T-cadherin were examined in HCT116 cells treated with adiponectin.

**Results:**

Serum adiponectin concentrations in patients with advanced adenoma and CRC were lower than those in controls. Adiponectin mRNA was not detected in colonic tissue, whereas AdipoRs mRNA was lower in advanced adenoma and CRC than that in normal colon tissues. Immunohistochemical staining demonstrated that adiponectin was expressed in spindle-shaped cells of the subepithelial layer in normal colon tissues, whereas ill-defined overexpression of adiponectin was seen in the stroma of advanced adenoma and CRC tissues. AdipoRs expression was strong in normal epithelium, but weak to negative in the epithelia of CRC tissues. Adiponectin downregulated COX-2 mRNA expression *in vitro,* but upregulated T-cadherin in HCT116 cells.

**Conclusions:**

Systemic adiponectin and local AdipoRs expression in the colon may be associated with anti-tumorigenesis during the early stages of CRC. These findings offer new insight into understanding the relationship between adiponectin and colorectal carcinogenesis.

**Electronic supplementary material:**

The online version of this article (doi:10.1186/1471-2407-14-811) contains supplementary material, which is available to authorized users.

## Background

Until quite recently, adipose tissue was regarded simply as an energy store. However, it is now considered to be an important endocrine organ, secreting a wide range of biologically active adipokines [1]. Adiponectin is the most abundant adipokine secreted from adipocytes; it is a 30-kDa adipokine composed of 244 amino acids [2–5]. Serum adiponectin level is inversely correlated with visceral obesity [5–8]. It is also known to have anti-diabetic, anti-inflammatory, and anti-atherosclerotic properties [2, 5, 9, 10]. Its action is mediated by binding to two main adiponectin receptors 1 (AdipoR1) and AdipoR2, and a third adiponectin receptor, T-cadherin. AdipoR1 is ex-pressed abundantly in skeletal muscle, whereas AdipoR2 is expressed predominantly in hepatocytes [5]. T-cadherin was identified as a receptor for the hexameric and high molecular weight species of adiponectin on vascular cells, not for the trimetric or globular species [11].

Epidemiological studies have consistently reported a significant link between obesity and malignancy [12–14]. Adiponectin is secreted mainly from adipocytes and has received considerable attention in relation to carcinogenesis in various cancers, including breast cancer, endometrial cancer, and hepatocellular carcinoma [15–17]. Several studies have demonstrated that adiponectin may have anti-proliferative properties in terms of carcinogenesis [8]. In addition, adiponectin reportedly suppresses cell proliferation in colorectal cancer cells. The effect of adiponectin has been evaluated in Caco 2, DLD 1, LoVo, SW480, and SW620 colorectal cancer cells but not in HCT119 and HT29 cells [8, 18, 19], although one study showed that adiponectin stimulates proliferation in HT29 cells [20].

However, the role of adiponectin in colorectal carcinogenesis in humans has not been established. In a prospective study, serum adiponectin concentration was associated with the risk of colorectal cancer (CRC) in males [21]. However, no association between serum adiponectin and CRC was observed in another study [22].

No study has compared the tissue expression patterns of adiponectin in normal-adenoma-carcinoma sequence of the colon, although differences in the expression of the two AdipoRs between normal colon epithelium and CRC have been reported [23, 24]. Whether the effects of adiponectin in colorectal carcinogenesis are indirect, through mediators such as insulin and inflammatory factors, and/or direct, through actions on cancer cells is also unclear [23]. Only one study has suggested that AdipoR1 negatively regulates the epithelial-mesenchymal transition (EMT) in breast cancer [25].

Thus, we investigated changes in adiponectin and AdipoRs in the normal-adenoma-carcinoma sequence of the human colon. We also assessed the direct influence of adiponectin on AdipoRs and EMT regulators including E-cadherin, and cyclooxygenase-2 (COX-2), and T-cadherin as a tumor suppressor, and not only a third adiponectin receptor, in colon cancer cells.

## Methods

### Study population

Tissue samples were obtained from 39 patients with CRC who underwent surgical resection or endoscopic removal, 29 patients with colorectal advanced adenoma (≥2 cm) who underwent polypectomies, and 10 healthy subjects with no colorectal pathological signs as a control group from a single institute between 2009 and 2011 (Table 1). Colonoscopy was performed on each subject in the morning before undergoing any surgical or endoscopic treatment, and at least two pieces of tissue for quantitative real-time polymerase chain reaction (qRT-PCR) were taken from the lesion and macroscopically normal mucosa. Additionally, two or three pieces of tissue was taken during colonoscopy for immunohistochemical staining in the control group, whereas surgical tissue sections were obtained after operations or endoscopic procedures in the advanced adenoma and CRC groups.

**Table 1:** **Clinicopathologic characteristics of the total study population**

	Control	Advanced adenoma	CRC	*p*value
	(n = 10)	(n = 29)	(n = 39)	
Age, years				0.091
Mean ± SD	54.5 ± 7.6	54.5 ± 7.6	60.2 ± 11.5	
Gender (%)				0.108
Female	4 (40.0)	5 (17.2)	16 (41.0)	
Male	6 (60.0)	24 (82.8)	23 (59.0)	
Body mass index, kg/m^2^				0.811
Mean ± SD	22.8 ± 2.4	23.9 ± 3.8	23.5 ± 2.8	
Location (%)				0.930
Cecum		1 (3.4)	1 (2.6)	
Ascending colon		4 (13.8)	6 (15.3)	
Hepatic flexure		2 (6.8)	1 (2.6)	
Transverse colon		4 (13.8)	4 (10.3)	
Descending colon		1 (3.4)	3 (7.8)	
Sigmoid colon		7 (20.8)	15 (38.4)	
Rectum		10 (38.0)	9 (23.0)	
TNM Stage (%)				
0 (Carcinoma *in situ*)			11 (28.2)	
I			6 (15.4)	
II			4 (10.3)	
III			16 (41.0)	
IV			2 (5.1)	

The medical records of each subject were reviewed for the following information: age, gender, body mass index (BMI), co-morbidity, presence or absence of diabetes mellitus, and pathological characteristics, such as location of cancer, and TNM stage. TNM staging was assessed according to the 7^th^ edition of the American Joint Committee on Cancer stage classification system [25]. BMI was calculated as weight divided by height^2^ (kg/m^2^) at the time of tissue sampling. According to the International Obesity Task Force for Asian adults [26], the normal weight and overweight groups were divided by a BMI cut-off of 23 kg/m^2^.

Participants with diabetes mellitus, BMI ≥30 kg/m^2^, or a severe accompanying disease, such as another cancer except CRC, major organ insufficiency, infectious disease, and inflammatory bowel disease were excluded.

All included patients provided written informed consent. This study was approved by the Institutional Review Board of Ewha Womans University MokDong Hospital (ECT205-9).

### Measurement of serum adiponectin

Blood samples were obtained from all subjects at the time of diagnosis in the morning. The Quantikine Human Adiponectin assay (R&D Systems, Minneapolis, MN, USA) was used to measure adiponectin levels. All assays were performed in duplicate. The normal range of adiponectin was defined between 855.5-21,424 ng/mL (mean, 8,841) according to the manufacturer’s data.

### Measurement of tissue adiponectin and AdipoRs mRNA expression

Total RNA was extracted from biopsy tissues using the easy-BLUE (iNtRON Biotechnology, Sungnam, Korea) total RNA extraction kit. The amount and purity of extracted RNAs were determined using spectrophotometry. Complementary DNA samples were prepared by reverse transcription using 2 μg RNA in each sample. The full-length adiponectin and globular adiponectin mRNA expression was carried out using SYBR Green Master Mix (Applied Biosystems, Foster City, CA, USA). The primers were: 5′-CTGGTGAGAAGGGTGAGAAA-3′ (forward) and 5′-CTTCTTGAAGAGGCTGACCT-3′ (reverse) for full-length adiponectin, and 5′-AACATGCCCATTCGCTTTAC-3′ (forward) and 5′-ATTACGCTCTCCTTCCCCAT-3′ (reverse) for globular adipo-nectin.

qRT-PCR for the AdipoRs was carried out using TaqMan Expression Master Mix (Applied Biosystems). The AdipoRs primers and probes were: the FAM-labeled Adiponectin Receptor1 (assay ID Hs00360422_m1) was used for AdipoR1, and the FAM-labeled Adiponectin Recptor2 (assay ID Hs00226105_m1) was used for AdipoR2. The geometric mean of two validated reference genes, including GAPDH (assay ID Hs99999905_m1) and β2 microglobulin (assay ID Hs99999907_m1), was used to adjust the expression of AdipoR1 and AdipoR2 in the qRT-PCR analysis. qRT-PCR was performed using a Real-Time 7000 system (Applied Biosystems). A non-template control was included in each experiment. All patient samples and non-template controls were assayed in duplicate.

### Measurement of adiponectin and AdipoRs protein expression in tissues and cell lines

Tissues and cell lysates were prepared in ice-cold PRO-PREP (Intron Biotech) and cleared by centrifugation (12,000 rpm, 30 min, 4°C). The protein concentration in each sample was estimated using the Bradford assay. Each protein sample (25 μg) was resolved on 10% sodium dodecyl sulphate-polyacrylamide gel electrophoresis, and transferred to a polyvinylidene fluoride membrane. The membrane was incubated for 1 h in blocking solution (5% non-fat milk in PBST) and incubated with primary antibodies for 3 h at room temperature. Primary antibodies included those against adiponectin (1:500; R&D Systems) and GAPDH (1:10,000; Fitzgerald Industries International Inc., Westborough, MA, USA). The blots were washed in PBST and incubated with HRP-conjugated secondary antibodies for 1 h at room temperature. Results were visualized using an enhanced chemiluminescent system (Invitrogen, Carlsbad, CA, USA).

### Immunohistochemical staining for adiponectin and AdipoRs in tissues

Immunohistochemistry was performed using the biotin-streptavidin method with the LSAB Kit (Dakocytomation, Carpentaria, CA, USA). Paraffinized tissues were sectioned at 4 μm thickness and deparaffinized with xylene twice for 5 min. The tissues were dehydrated in an ethanol series (100-70%) for 5 min each, and washed with distilled water. Antigene retrieval conditions included heat-induced epitope retrieval in 10 nM citrate buffer (pH 6.0) for 7 min.

Endogenous peroxidase activity was blocked by incubating the sections in 3% hydrogen peroxide solution for 5 min. The tissues were incubated with the monoclonal anti-adiponectin (1: 250; R&D Systems), AdipoR1 (1:700; Phoenix Pharmaceutical, Burlingame, CA, USA), and AdipoR2 (1:300; Phoenix Pharmaceutical) in a moist chamber for 1 h at room temperature. The negative control was incubated with diluent and background reducing components (ready-to-use, Dakocytomation) instead of primary antibody. Subsequently, the tissues were incubated for 30 min with a biotinylated immunoglobulin secondary antibody solution. The tissues were then incubated with streptavidin peroxidase solution for 30 min, and a liquid DAB substrate chromogen solution was used as a substrate to yield the brown-colored reaction products. Hematoxylin staining was performed as a counter stain. The stained tissues were photographed using an Olympus microscope with ColorView3 digital camera (Soft Imaging system, Gebh, Germany).

Adiponectin and AdipoRs expression were graded in terms of density and distribution to analyze the data. Staining of epithelial cells was classified as none, mild, moderate, or intense. The expression distribution was quantified as the percentage of positive epithelial cells and assigned to one of the following four categories: <10%, 10-30%, 30-60% or >60%.

Each lesion was examined and scored independently by a reviewer blinded to the patient information. Every suspected pathological result was reviewed by a pathologist.

### Cell culture

HCT116, HT29, and CCD-18Co cells were obtained from the Korean Cell Line Bank (KCLB, Seoul, Korea). The cell lines were cultured in RPMI 1640 or DMEM. All media were supplemented with 10% fetal bovine serum (FBS) (v/v) and 1% penicillin/streptomycin. Cell cultures were incubated in a CO_2_ incubator with a humidified atmosphere of 95% air/5% CO_2_ at 37°C. Cultures generally reached 80-90% confluence at 2–4 days after seeding. After they reached confluence, the cells were subcultured (split ratio 1:4 to 1:5) using 0.25% trypsin-EDTA.

### Adiponectin treatment of the cell lines

HCT116 cells were plated in 12-well tissue culture dishes at a density of 1 × 10^5^ or 2 × 10^5^ per well. The cells were allowed to adhere overnight and were treated with recombinant human full-length adiponectin (BioVendor Laboratory Medicine, Modrice, Czech Republic) at 0.1, 1, or 20 μg/mL in FBS-containing medium for 24 h. Subsequently, the medium was discarded, and the cells were washed with PBS before RNA extraction.

### Measurement of AdipoRs, E-cadherin, COX-2, and T-cadherin mRNA expression in adiponectin-treated cells

Total RNA was extracted from HCT116 cells using the easy-BLUE (iNtRON Biotechnology) total RNA extraction kit. The amount and purity of the extracted RNAs were determined by spectrophotometry. Complementary DNA samples were prepared by reverse transcription using 2 μg RNA per sample. qRT-PCR was carried out using the TaqMan Expression Master Mix (Applied Biosystems) for AdipoRs. The primers used were as follows: the FAM-labeled Adiponectin Receptor1 (assay ID Hs00360422_m1) was used for AdipoR1 and the FAM-labeled Adiponectin Recptor2 (assay ID Hs00226105_m1) was used for AdipoR2. qRT-PCR was carried out using the SYBR Green Master Mix (Applied Biosystems) for E-cadherin, COX-2, and T-cadherin. The primers used were: 5′-TGGGTTATTCCTCCCATCAG-3′ (foward) and 5′-TTTGTCAGGGAGCTCAGGAT-3′ (reverse) for E-cadherin, 5′-TTCAAATGAGATTGTGGAAAAAT-3′ (forward) and 5′- AGATCATCTCTGCCTGAGTATCTT-3′ (reverse) for COX-2 and 5′- GATGTTGGCAAGGTAGTCGAT-3′ (forward) and 5′-GCTCCCTGTGTTCTCATTGAT-3′ (reverse) for T-cadherin. Expression levels were presented as a ratio to GAPDH (assay ID Hs99999905_ml) as the internal control. qRT-PCR was performed using a Real-Time 7000 system (Applied Biosystems). A non-template control was included in each experiment. All patient samples and non-template controls were assayed in duplicate.

### Statistical analyses

All statistical calculations were performed using SPSS ver. 18.0 software (SPSS, Inc., Chicago, IL, USA). Continuous data are presented as mean ± standard deviation. Categorical data are presented as percentages. Differences in serum adiponectin concentrations and AdipoRs mRNA expression levels among groups were evaluated using the Mann–Whitney *U*-test because of a skewed distribution. Differences in AdipoRs mRNA expression levels between the lesion and their matched normal colon tissues in the same patient with advanced adenoma or CRC were analyzed using the repeated paired *t*-test. Immunohistochemical staining results were analyzed comparatively using linear associations. Repeated-measures analysis of variance with a *post hoc* Tukey’s multiple comparison test was used to calculate the differences among the means of the *in vitro* assay groups. A *p* value <0.05 was considered significant.

## Results

### Serum adiponectin levels in advanced adenoma and CRC were significantly lower than those in the control group, regardless of BMI

**Figure 1:**
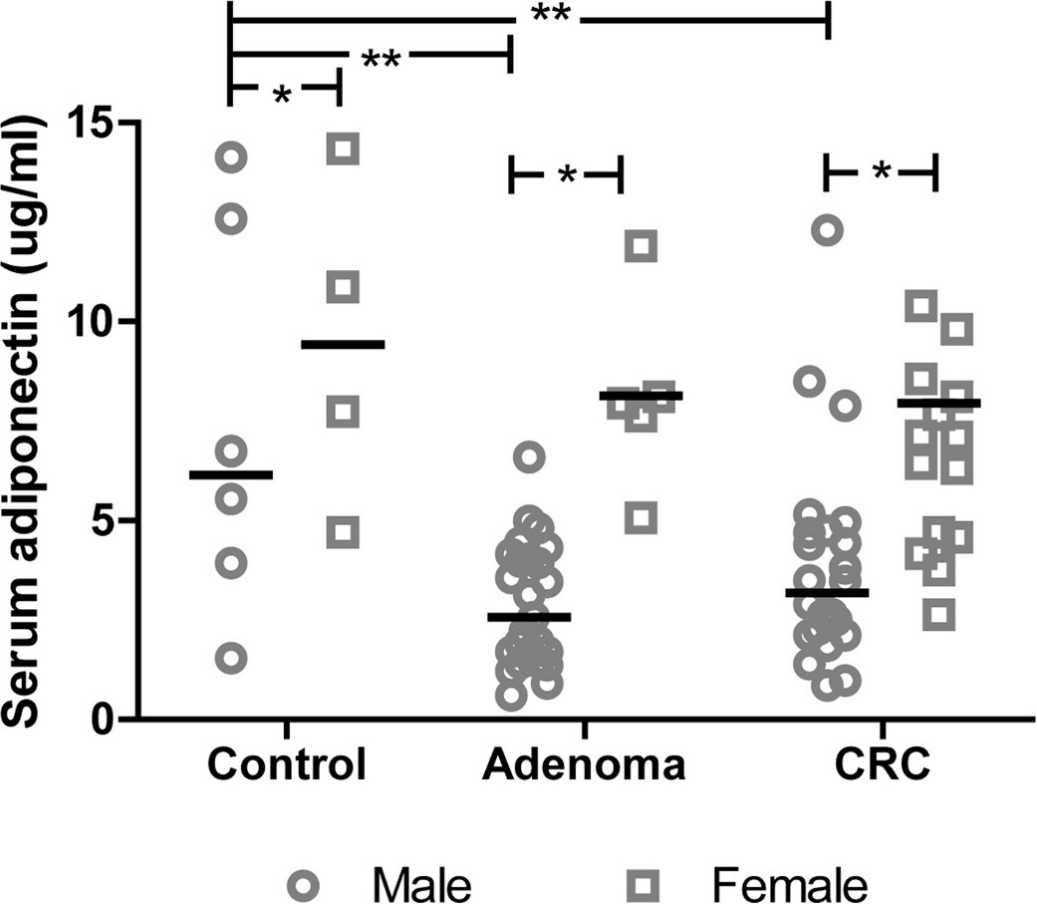
**Serum adiponectin concentration according to gender in controls, and patients with advanced adenoma and CRC.** Significant gender differences were seen for each lesion. Serum adiponectin concentrations in males with advanced adenoma and CRC were significantly lower than those in the control group. No significant differences in serum adiponectin concentration were observed among the groups in females. * *p* <0.05; ** *p* <0.001.

As shown in Figure 1, serum adiponectin concentrations varied according to gender. Serum adiponectin concentrations in females were higher after comparing each subgroup between males and females (*p* <0.05). Serum adiponectin concentrations in males with advanced adenoma and CRC were significantly lower than those of the control group (*p* <0.001). However, serum adiponectin concentrations in patients with CRC were not significantly different from those in patients with advanced adenoma. No significant difference in serum adiponectin concentrations was observed among female controls or those with advanced adenoma or CRC.

Because there was a significant inverse correlation between serum adiponectin concentrations and BMI in males (*r* = -0.282, *p* = 0.016), we analyzed serum adiponectin concentrations according to BMI subgroups; *i.e*., above and below the 23 kg/m^2^ cut off (see Additional file 1). Serum adiponectin concentrations in the control subgroup with BMI >23 kg/m^2^ were lower than those in the subgroup with a BMI <23 kg/m^2^ (*p* <0.001). However, no significant differences in serum adiponectin concentrations were observed according to BMI in the advanced adenoma and CRC groups.

### Adiponectin in colon tissues originates from adipose tissues through systemic flow and the adiponectin expression pattern changes in the normal-adenoma-carcinoma sequence

**Figure 2:**
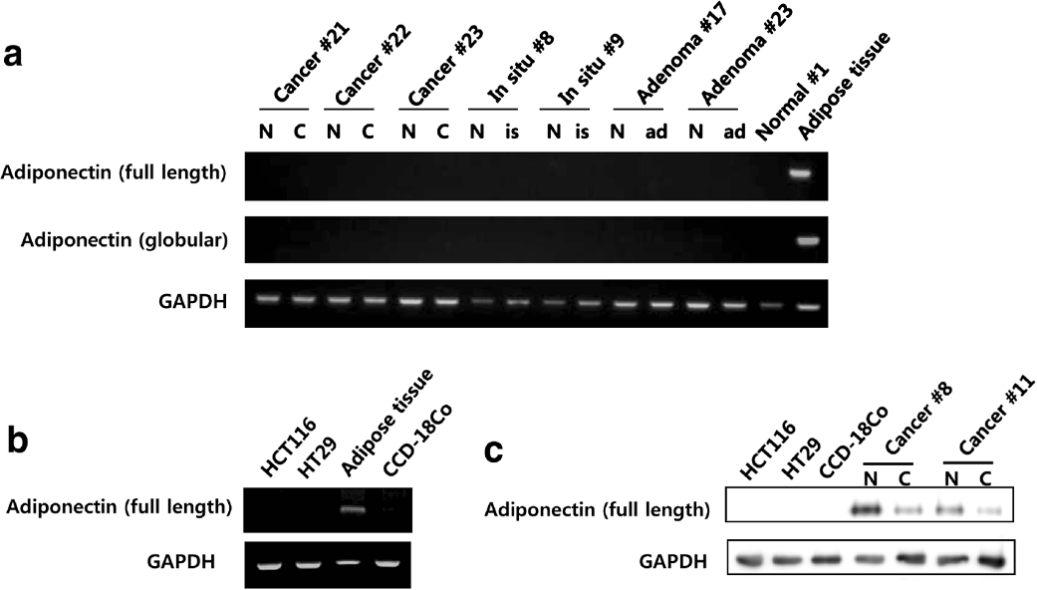
**Adiponectin mRNA and protein levels in cell lines and tissues. (a)** Adiponectin mRNA expression was not detected by RT-PCR in tissues, except for adipose tissue of controls. **(b)** Adiponectin mRNA expression was not detected by RT-PCR in CRC cell lines, except for the adipose tissue of controls. **(c)** Adiponectin protein was detected by Western blotting in CRC and normal colon tissues, but not in cell lines. N, normal colon tissue; C, colorectal cancer; is, carcinoma *in situ*; ad, advanced adenoma.

To determine whether adiponectin originated from external adipose tissues through systemic flow or local production, we measured adiponectin mRNA and protein levels in colon tissues from each group and adipose tissue as a control, as well as in HCT116 and HT29 cells as a CRC cell line, and CCD-18Co cells as an embryonic colon fibroblast cell line. Adiponectin mRNA was not detected in any colon tissue or cell lines but was detected in control adipose tissue (Figure 2a, 2b). Furthermore, adiponectin protein was detected in both CRC and normal colon tissues, but not in the respective cell lines (Figure 2c). Thus, these findings suggest that adiponectin in CRC and normal colon tissue is of systemic origin and is not produced locally. This result explains why neither adiponectin mRNA nor protein was detected in the cell lines.

**Figure 3:**
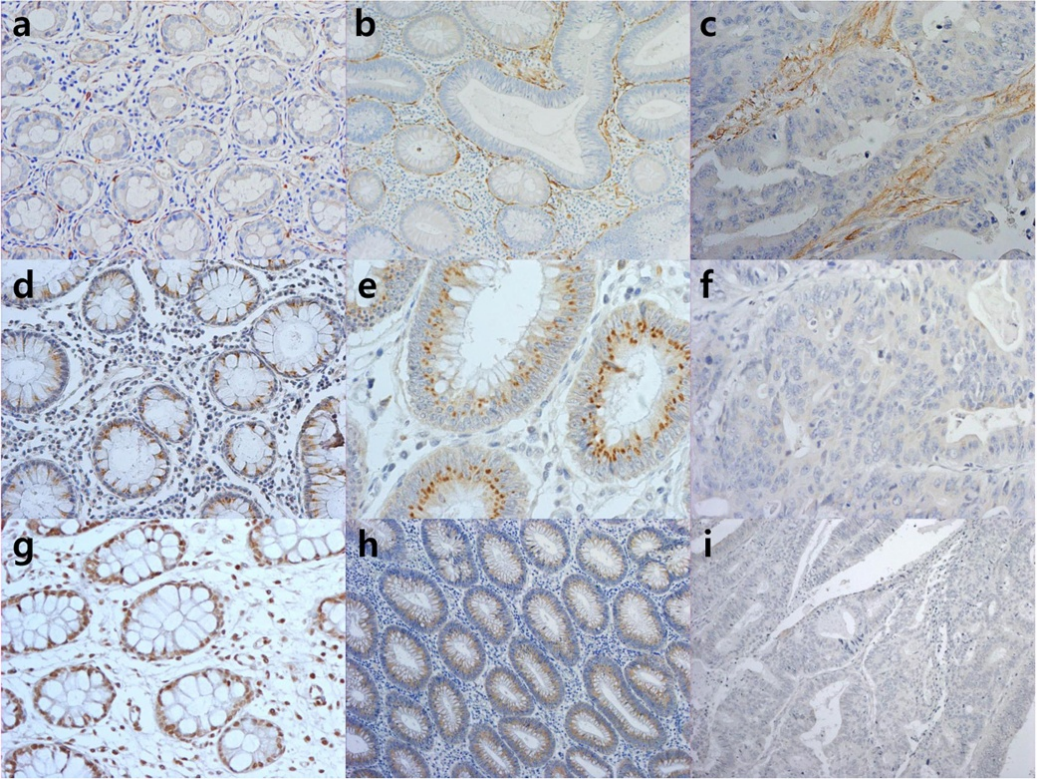
**Immunohistochemical staining of adiponectin, AdipoR1, and AdipoR2. (a–c)** Localization of adiponectin staining in normal colon tissue (**a**, ×200), advanced adenoma (**b**, ×200) and CRC (**c**, ×200) differed from spindle-shaped cells in the subepithelial area to a diffuse area in the lamina propria. **(d–i)** Staining for AdipoR1 (**d**, ×200) and AdipoR2 (**g**, ×200) was positive in the epithelium of normal colon tissues. AdipoR1 (**e**, ×400) and AdipoR2 (**h**, ×100) showed moderate staining intensities in the epithelial layer of advanced adenoma. In CRC, AdipoR1 (**f**, ×400) and AdipoR2 (**i**, ×100) showed weak or no staining.

Immunohistochemical staining for adiponectin was performed to compare the adiponectin expression patterns among normal colon, advanced adenoma, and CRC tissues. Adiponectin expression in normal colon tissues was predominantly positive in the subepithelial mesenchymal cells of the lamina propria but not in the colonic epithelial cells. The expression had an appearance similar to the spindle-shaped cells that run parallel to the lining of the epithelium (Figure 3a). However, the adiponectin expression pattern of the thin layer in the subepithelial area was obliterated, while an indeterminate over-expression pattern in stroma was seen in 71.4% of advanced adenoma and 97.4% of CRC tissues (Figure 3b, c).

### AdipoRs expression in epithelium decreased in the progression from normal tissue to advanced adenoma

**Figure 4:**
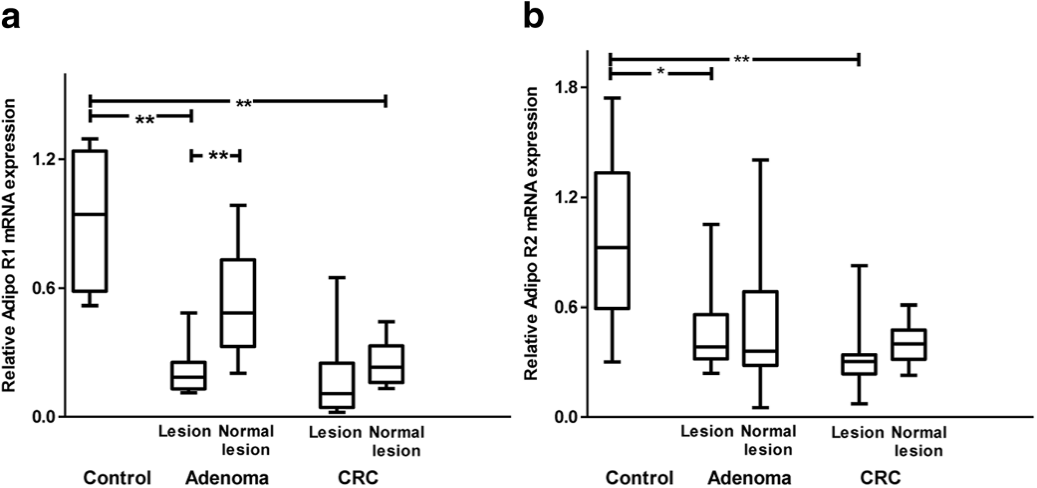
**mRNA expression of AdipoR1 and AdipoR2 in controls and patients with advanced adenoma and CRC. (a, b)** AdipoR1 and AdipoR2 mRNA expression levels in advanced adenoma and CRC tissues were significantly lower than those in the control group. AdipoRs mRNA expression levels were not significantly different between advanced adenoma and the CRC tissues. The top and bottom of the boxes are the 25^th^ and 75^th^ percentiles. The difference between the quartiles is the interquartile range. The line across the middle of the box shows the median value. * *p* <0.05, ** *p* <0.001.

We examined AdipoR1 and AdipoR2 mRNA and protein expression levels in specimens from control, advanced adenoma, and CRC tissues by qRT-PCR and immunohistochemical staining. As shown in Figure 4, AdipoR1 and AdipoR2 mRNA expression levels were significantly lower in the advanced adenoma and CRC groups than those in the controls. However, no difference in AdipoRs mRNA expression levels was observed between the advanced adenoma and CRC groups.

**Figure 5:**
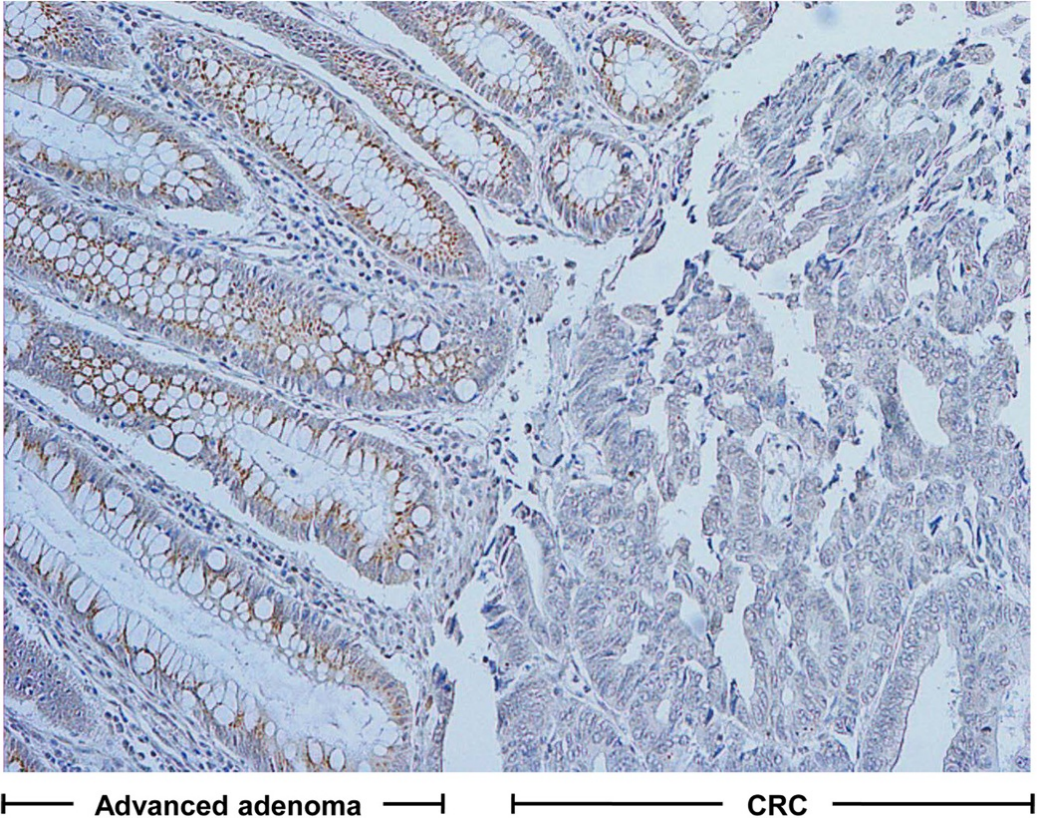
**Representative examples of AdipoR1 immunohistochemical staining with carcinoma in situ arising from advanced adenoma (×100).**

AdipoR1 and AdipoR2 expression by immunohistochemical staining were localized in the colon epithelium and was positive in all (100%) of the control and advanced adenoma tissues and in 62.8% and 88.5% of CRC tissues. The distribution and density of staining for AdipoRs decreased significantly in the order of normal to advanced adenoma to CRC tissues (Tables 2, 3; Figure 3d–i). We confirmed these differences in AdipoR1 expression between advanced adenoma and CRC in nine patients with carcinoma *in situ*, which arose from advanced adenoma (*p* = 0.034, Wilcoxon’s signed-rank test), as shown in Additional file 2 and Figure 5. No significant differences were observed in the relationship between AdipoRs staining and the clinicopathological characteristics of CRC such as differentiation, TNM stage, or tumor location.

**Table 2:** **Immunohistochemical staining scores for AdipoR1**

	Control	Advanced adenoma	CRC	*p*value
	(n = 10)	(n = 29)	(n = 35)	
Density (%)				<0.001
None	0	0	13 (37.2)	
Mild	0	6 (20.7)	14 (40.0)	
Moderate	2 (20.0)	8 (27.6)	6 (17.1)	
Intense	8 (80.0)	15 (51.7)	2 (5.7)	
Distribution (%)				<0.001
< 10%	0	0	13 (37.1)	
10–30%	0	1 (3.4)	0 (0.0)	
30–60%	0	6 (20.7)	3 (8.6)	
> 60%	10 (100.0)	22 (75.9)	19 (54.3)	

CRC, colorectal cancer.

**Table 3:** **Immunohistochemical staining scores for AdipoR2**

	Control	Advanced adenoma	CRC	*p*value
	(n = 10)	(n = 14)	(n = 26)	
Density (%)				<0.001
None	0	0	3 (11.5)	
Mild	0	0	11 (42.4)	
Moderate	2 (20.0)	8 (57.1)	9 (34.6)	
Intense	8 (80.0)	6 (42.9)	3 (11.5)	
Distribution (%)				0.027
< 10%	0	0	3 (11.5)	
10–30%	0	0	0	
30–60%	0	0	0	
> 60%	10 (100.0)	14 (100.0)	23 (88.5)	

CRC, colorectal cancer.

### Adiponectin downregulates COX-2 mRNA expression and upregulates T-cadherin mRNA expression in HCT116 cells

**Figure 6:**
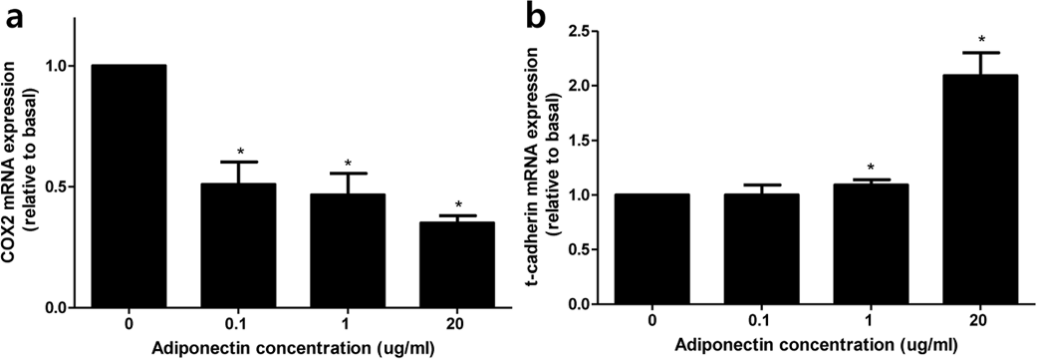
**Influence of adiponectin on COX-2, T-cadherin in HCT116 cells. (a)** Adiponectin downregulated COX-2 mRNA expression in a dose-dependent manner. **(b)** Expression of T-cadherin mRNA was upregulated by adiponectin in a dose-dependent manner. * *p* <0.05 *vs*. control.

Based on the changes in the adiponectin expression pattern in subepithelial area and AdipoRs expression pattern in the colonic epithelium in the normal-adenoma-carcinoma sequence, we investigated the direct effects of adiponectin on AdipoRs and EMT-associated regulators on CRC development, progression and metastasis. The mRNA expression levels of AdipoRs, E-cadherin, and COX-2 as EMT regulators, and T-cadherin, not only as a tumor suppressor but also as a co-receptor of adiponectin, were examined in HCT116 cells treated with adiponectin. Adiponectin treatment downregulated COX-2 mRNA expression, upregulated T-cadherin mRNA expression in a dose-dependent manner (Figure 6), and 20 μg/mL adiponectin slightly upregulated AdipoR2 mRNA expression in HCT116 cells (see Additional file 3). Adiponectin treatment had no effect on E-cadherin mRNA expression levels in HCT116 cells (see Additional file 4).

## Discussion

Our findings suggest several possible mechanisms for the effect of adiponectin on the early stage of colorectal carcinogenesis. Adiponectin has anti-proliferative and anti-tumorigenesis properties. A deficiency of adiponectin seems to be associated with the development of an early neoplasm, rather than advanced CRC. Serum adiponectin concentrations were significantly lower in male patients with advanced adenoma than those in healthy control males. However, no difference in serum adiponectin concentrations were observed between patients with advanced adenoma and CRC. These results are consistent with studies on the anti-tumorigenesis properties of adiponectin, which show that low serum adiponectin concentrations are strongly associated with an increased risk of colorectal adenoma or early CRC, but not with advanced CRC [27–29]. Additionally, the gender difference in adiponectin concentrations seen in our patients has been reported previously [27, 30–34]. This result is consistent with the report by Wei *et al*. that males with low plasma adiponectin levels have a higher risk or CRC than those with higher levels [21].

A second possible mechanism for the role of adiponectin in colorectal neoplasm is a direct anti-tumorigenic effect. Interest in the association between adiponectin and CRC originated from consideration of CRC as a complication of obesity [35, 36]. Many reports have suggested that adiponectin affects cancer development indirectly, via insulin resistance [33, 35, 37, 38]. We compared serum adiponectin concentrations among the groups after stratification by BMI, based on the inverse correlation between obesity and serum adiponectin concentrations. Contrary to our expectations, serum adiponectin concentrations were lower in patients with colorectal neoplasms regar-dless of the presence of obesity, and did not differ significantly between the overweight and normal weight subgroups in the advanced adenoma and CRC groups. This finding was similar to findings of previous studies, which showed a link among obesity, CRC, and adiponectin [39]. This finding suggests that adiponectin could exert local effects on colon tissues [24, 38]. We also found that adiponectin downregulated COX-2 mRNA expression and upregulated T-cadherin mRNA expression in colon cancer cells in a dose-dependent manner; thus, suggesting direct tumor suppression. These findings support previous reports that adiponectin directly suppresses proliferation of colon cancer cells [40, 41]. Moreover, studies have demonstrated a probable direct inhibitory effect of adiponectin in colorectal carcinogenesis. Fujisawa et al. and Byeon et al. demonstrated the inhibition of CRC cell growth by adiponectin using the MTT assay [42, 43]. These reports suggest another role of adiponectin, in addition to its indirect role in hormone modulation in the metabolic environment. It is possible that adiponectin originating from adipose tissues influences colonic tissue via systemic blood flow. We did not detect mRNA expression of adiponectin in any colon tissue, HCT116, and HT29 cancer cells or CCD-18Co embryonic colon myofibroblast cells, despite the positive results in the control adipose tissue. However, adiponectin was detected in colon tissue, but not in colonic epithelial cells, by Western blotting and immunohistochemical staining.

Adiponectin expression by immunohistochemical staining was localized to spindle-shaped cells of the subepithelial thin layer surrounding each epithelial nest in normal colon tissue. Adiponectin expression in advanced adenoma and CRC tissues was seen in the stroma with an ill-defined pattern. Considering their location and morphology, adiponectin-expressing cells were likely myofibroblasts [44–46]. Although CCD-18Co cells did not express adiponectin as determined by Western blotting, it is possible that the spindle-shaped cells are myofibroblasts because the CCD-18Co cell line originated from an embryo. Myofibroblasts adjacent to neoplastic cell nests express important molecules that regulate the EMT to cancer progression [46]. Thus, the association between adiponectin and EMT regulators was investigated to determine the pathway that mediates the direct role of adiponectin in colorectal carcinogenesis. Among the well-known colonic EMT regulators, COX-2, which originates mainly from stromal myofibroblasts surrounding the CRC nest, and E-cadherin, which is negatively regulated by COX-2 in the EMT and metastasis, were examined [45, 47]. T-cadherin, considered a tumor suppressor in colon, and a third adiponectin receptor has also been investigated [48–50].

The *in vitro* assay and immunohistochemical staining results suggest that adiponectin may prevent colorectal carcinogenesis and proliferation by downregulating COX-2 expression. This result may be associated with recent reports that adiponectin directly inhibits colon cancer cell proliferation via AdipoR1- and AdipoR2-mediated AMP-activated protein kinase activation [40]. However, the role of the AMP-activated protein kinase pathway in the regulation of COX-2 expression in colon cancer cells should be investigated further [51].

Some studies have suggested that AdipoRs expression is greater in cancerous colonic tissues, with a compensatory mechanism in the decline in serum adiponectin concentrations [37, 52]. Other studies have shown that AdipoRs expression is not different between control and colon cancer specimens [23]. However, AdipoRs expression in the epithelium decreased in the progression from normal tissue to advanced adenoma in both qRT-PCR and immunohistochemical staining in our study. This result demonstrates that changes in AdipoRs expression may mediate the effects of adiponectin according to the normal-adenoma-carcinoma sequence [39, 42]. It seems that a large population-based study is required to examine these discrepancies.

This study had some limitations. Given the small size of the study population, our findings should not be generalized. However, we controlled for the effects of various confounding factors, such as metabolic syndrome, medications, and obesity. We were unable to determine an association between clinicopathological CRC data and adiponectin or AdipoRs expression due to the small sample size. Despite these limitations, this study is important because it suggests a hypothesis for the roles of adiponectin and AdipoRs during the early stages of colorectal carcinogenesis.

## Conclusions

In conclusion, serum adiponectin concentrations and AdipoRs expression in colon tissues change reciprocally in the normal-adenoma-carcinoma progression. Decreased AdipoRs expression in the colonic epithelium according to colorectal neoplasm progression was accompanied by increased or modified adiponectin expression in the mesenchyme. The increased adiponectin concentration leads to downregulation of COX-2 mRNA expression and upregulation of T-cadherin mRNA expression. These findings offer new insights into the relationship between adiponectin and colorectal carcinogenesis.

## Electronic supplementary material

Additional file 1: **Serum adiponectin concentration according to BMI in controls, and patients with advanced adenoma and CRC.** Serum adiponectin concentration according to BMI in males. In the control group, the subgroup with BMI >23 kg/m^2^ had lower serum adiponectin concentrations than the subgroup with BMI <23 kg/m^2^. No significant difference was observed between the BMI subgroups in patients with advanced adenoma and CRC. Line indicates the median serum adiponectin concentration. * *p* <0.05; ** *p* <0.001. (TIFF 61 KB)

Additional file 2: **Differences in AdipoR1 expression between patients with advanced adenoma and CRC in nine patients with carcinoma** ***in situ*** **that arose from advanced adenoma.** (PDF 73 KB)

Additional file 3: **Influence of adiponectin on AdipoRs in HCT116 cells.** (a) Adiponectin had no significant effect on the expression of AdipoR1 mRNA. (b) The expression of AdipoR2 mRNA was slightly, but significantly, up-regulated under 20 μg adiponectin treatment. * *p* <0.05 *vs*. control. (TIFF 311 KB)

Additional file 4: **Influence of adiponectin on E-cadherin levels in HCT116 cells.** Adiponectin had no significant effect on E-cadherin mRNA expression. (TIFF 247 KB)

## Abbreviations

AdipoRs: Adiponectin receptors
AdipoR1: Adiponectin receptor 1
AdipoR2: Adiponectin receptor 2
BMI: Body mass index
CRC: Colorectal cancer
EMT: Epithelial-mesenchymal transition
qRT-PCR: Quantitative real-time polymerase chain reaction.
