# Supplementary figures and images for: Involvement of adiponectin in early stage of colorectal carcinogenesis
Source: BMC Cancer. 2014 Nov 5;14:811. doi: 10.1186/1471-2407-14-811 (PMC4232655; doi:10.1186/1471-2407-14-811)

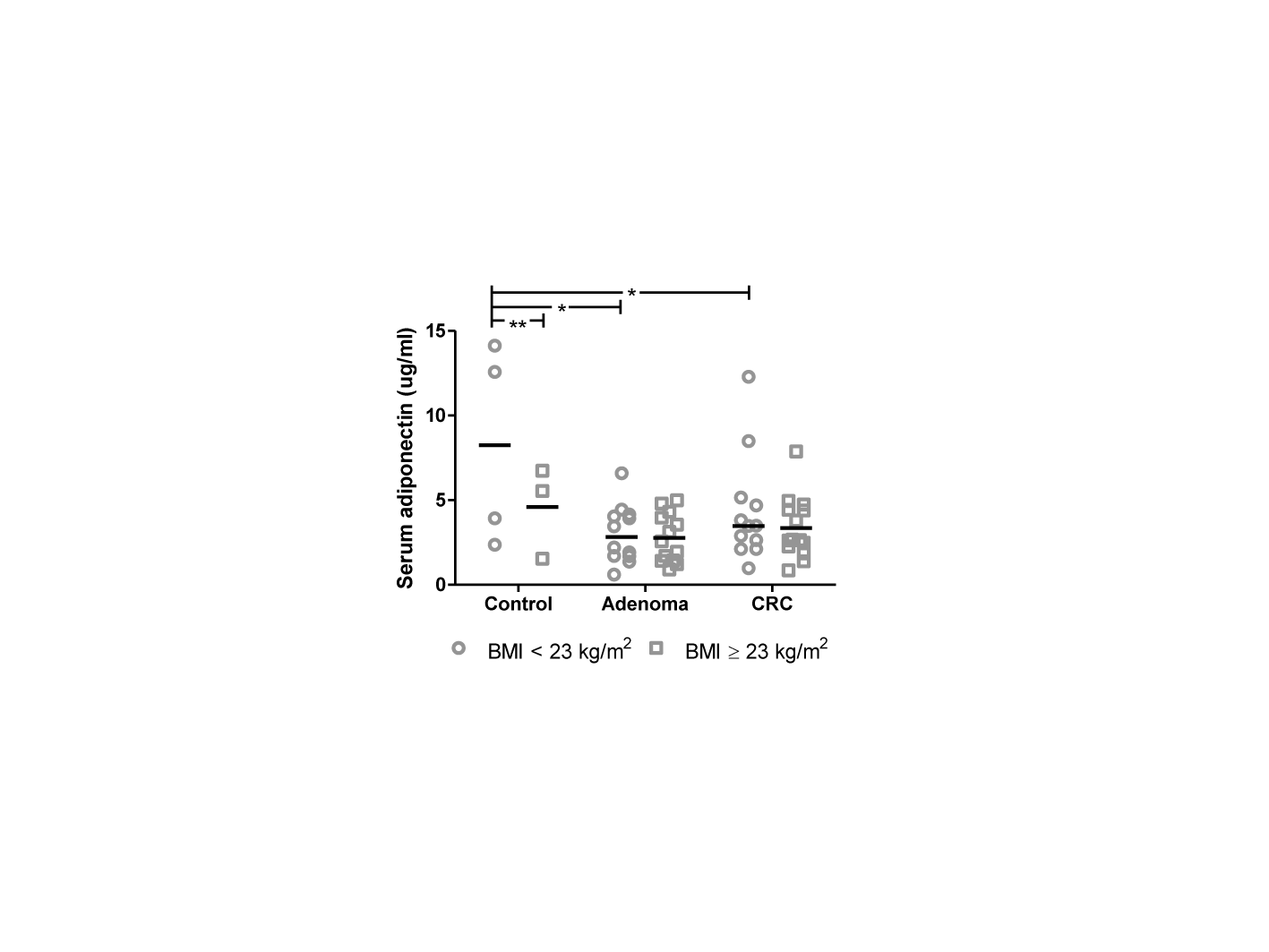

Supplement: Supplementary file 1 — Additional file 1: Serum adiponectin concentration according to BMI in controls, and patients with advanced adenoma and CRC. Serum adiponectin concentration according to BMI in males. In the control group, the subgroup with BMI >23 kg/m2 had lower serum adiponectin concentrations than the subgroup with BMI <23 kg/m2. No significant difference was observed between the BMI subgroups in patients with advanced adenoma and CRC. Line indicates the median serum adiponectin concentration. * p <0.05; ** p <0.001. (TIFF 61 KB) [file 12885_2013_4989_MOESM1_ESM.tiff]

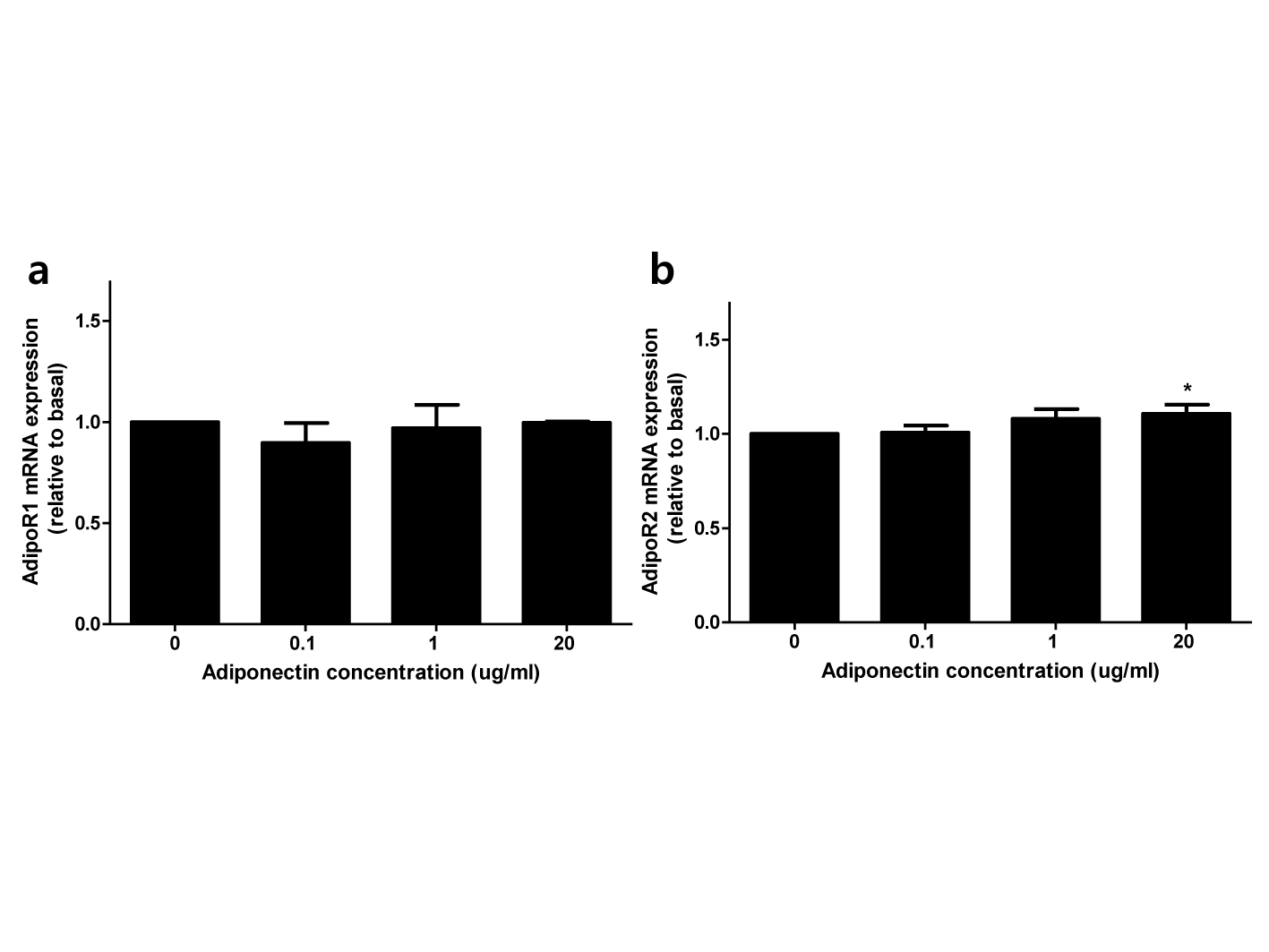

Supplement: Supplementary file 3 — Additional file 3: Influence of adiponectin on AdipoRs in HCT116 cells. (a) Adiponectin had no significant effect on the expression of AdipoR1 mRNA. (b) The expression of AdipoR2 mRNA was slightly, but significantly, up-regulated under 20 μg adiponectin treatment. * p <0.05 vs. control. (TIFF 311 KB) [file 12885_2013_4989_MOESM3_ESM.tiff]

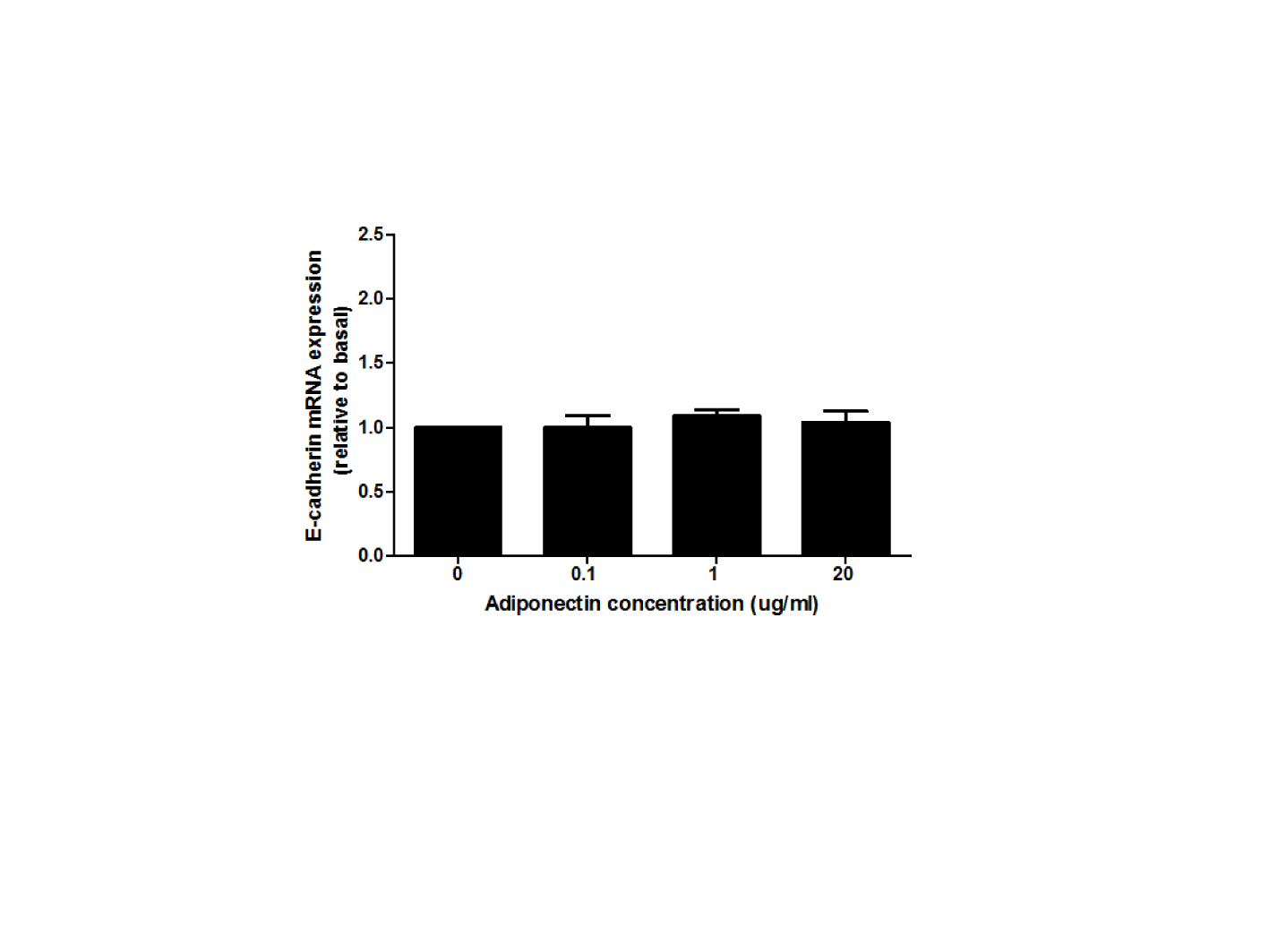

Supplement: Supplementary file 4 — Additional file 4: Influence of adiponectin on E-cadherin levels in HCT116 cells. Adiponectin had no significant effect on E-cadherin mRNA expression. (TIFF 247 KB) [file 12885_2013_4989_MOESM4_ESM.tiff]
