# Supplementary material for: Involvement of adiponectin in early stage of colorectal carcinogenesis
Source: BMC Cancer. 2014 Nov 5;14:811. doi: 10.1186/1471-2407-14-811 (PMC4232655; doi:10.1186/1471-2407-14-811)
Supplement: Supplementary file 2 — Additional file 2: Differences in AdipoR1 expression between patients with advanced adenoma and CRC in nine patients with carcinoma in situ that arose from advanced adenoma. (PDF 73 KB) [file 12885_2013_4989_MOESM2_ESM.pdf]

Additional file 2 - Differences in AdipoR1 expression between patients with advanced adenoma and CRC in nine patients with carcinoma *in situ* that arose from advanced adenoma

No. of Sample	Density of AdipoR1 expression		*p* value
	Advanced adenoma	CRC	
1	Moderate	None	
2	Moderate	Mild	
3	None	None	
4	Moderate	Mild	
5	None	None	0.034
6	None	None	
7	Moderate	Mild	
8	None	None	
9	Mild	None	
